# Supplementary material for: The biomarkers of key miRNAs and target genes associated with acute myocardial infarction
Source: PeerJ. 2020 May 13;8:e9129. doi: 10.7717/peerj.9129 (PMC7229769; doi:10.7717/peerj.9129)
Supplement: Table S1 [file peerj-08-9129-s002.docx]

**Supplement Table 1 |** The information of GEO database

|  | AMI sample count | Control sample count | Platforms |
| --- | --- | --- | --- |
| GSE24591 | 3 | 4 | Agilent-019118 Human miRNA Microarray 2.0 G4470B |
| GSE31568 | 20 | 70 | febit Homo Sapiens miRBase 13.0 |
