# Supplementary material for: The biomarkers of key miRNAs and target genes associated with acute myocardial infarction
Source: PeerJ. 2020 May 13;8:e9129. doi: 10.7717/peerj.9129 (PMC7229769; doi:10.7717/peerj.9129)
Supplement: Table S2 [file peerj-08-9129-s003.docx]

**Supplement Table 2 |** Characteristics of AMI patients and control group

| Characteristics | AMI group (n=11) | Healthy group (n=11) | *p*-value |
| --- | --- | --- | --- |
| Age (year) | 44.45±8.96 | 55.55±9.39 | 0.56 |
| Male/Female (n/n) | 4/7 | 6/5 | 0.39 |
| Smoking (%) | 54.55 | 36.36 | 0.67 |
| Diabetes (%) | 27.27 | 18.18 | 1.00 |
| Hypertension (%) | 54.55 | 27.27 | 0.39 |
| Total cholesterol (mmol/L) | 5.67±1.46 | 3.92±0.69 | 0.026* |
| Triglycerides (mmol/L) | 1.92±0.30 | 1.18±0.18 | 0.308 |
| HDL (mmol/L) | 0.84±0.38 | 1.53±0.27 | 0.63 |
| LDL (mmol/L) | 2.58±0.40 | 3.50±0.47 | 0.19 |
| Blood glucose (mmol/L) | 6.67±0.66 | 5.06±0.96 | 0.21 |
| CK-MB (U/L) | 35.91±20.17 | 16.00±3.50 | 0.008** |
| cTnT | 2.00±0.44 | 0.05±0.17 | <0.001** |
| NT-proBNP (pg/ml) | 243.18±106.07 | 58.44±25.27 | 0.006** |
