# Supplementary material for: The biomarkers of key miRNAs and target genes associated with acute myocardial infarction
Source: PeerJ. 2020 May 13;8:e9129. doi: 10.7717/peerj.9129 (PMC7229769; doi:10.7717/peerj.9129)
Supplement: Table S3 [file peerj-08-9129-s004.docx]

**Supplement Table 3 |** Relationships to conventional prognostic markers

|  | r | p-value |
| --- | --- | --- |
| miR-24-1* | -0.722 | <1*10^-3^  *** |
| miR-33a | -0.383 | 0.079 |
| miR-34a | -0.301 | 0.173 |
| miR-101 | -0.444 | 0.038 * |
| miR-139-3p | -0.425 | 0.048 * |
| miR-142-3p | -0.174 | 0.439 |
| miR-545 | -0.570 | 0.006 ** |
| miR-598 | -0.447 | 0.037 * |
