# Supplementary figures and images for: The biomarkers of key miRNAs and target genes associated with acute myocardial infarction
Source: PeerJ. 2020 May 13;8:e9129. doi: 10.7717/peerj.9129 (PMC7229769; doi:10.7717/peerj.9129)

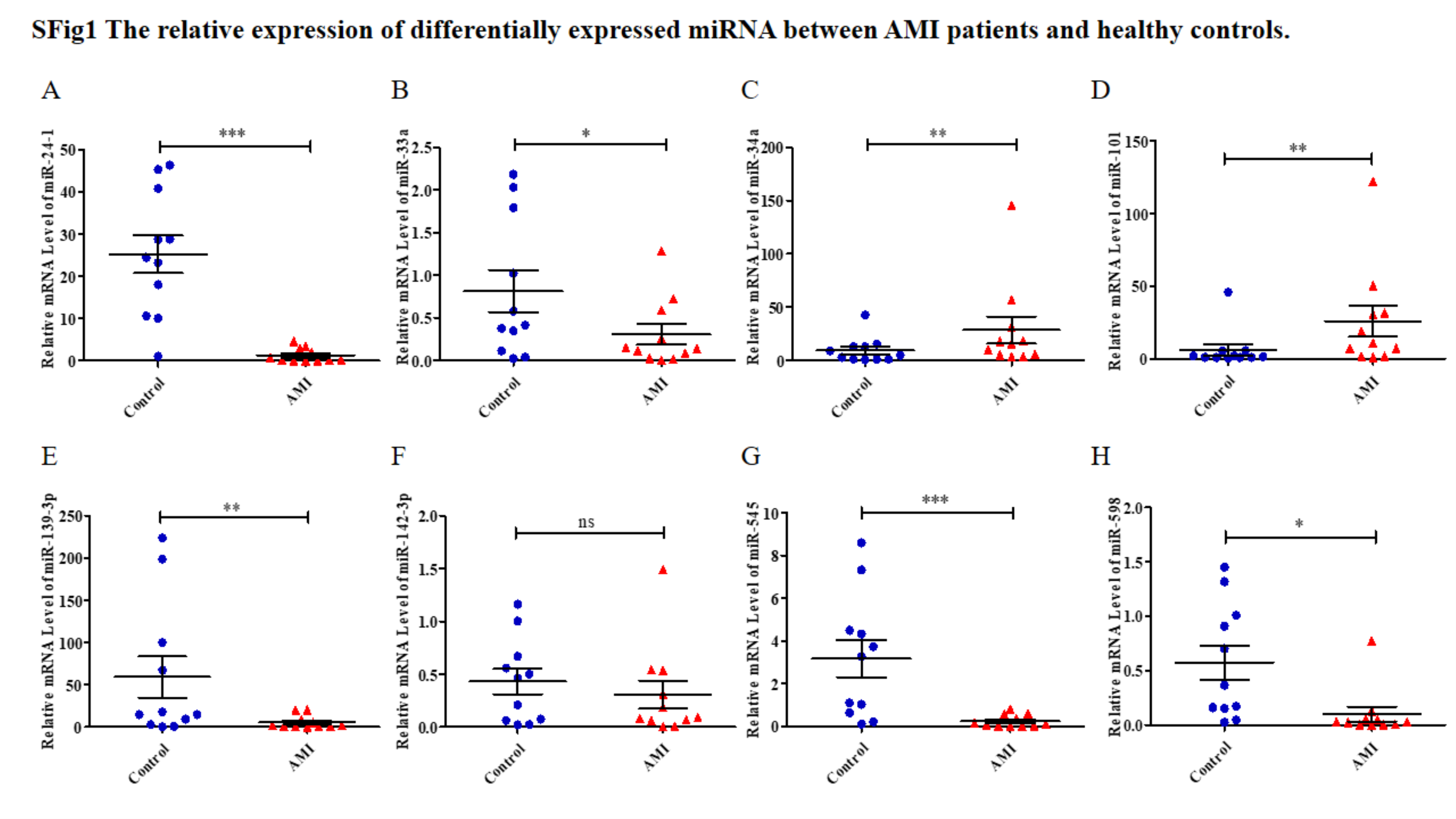

Supplement: Fig. S1 [file peerj-08-9129-s005.png]
